# Fibrin Biopolymer Incorporated with Antimicrobial Agents: A Proposal for Coating Denture Bases

**DOI:** 10.3390/ma14071618

**Published:** 2021-03-26

**Authors:** Helena Sandrini Venante, Ana Paula Chappuis-Chocano, Oscar Oswaldo Marcillo-Toala, Rafaela Alves da Silva, Rodrigo Moreira Bringel da Costa, Mariana Domingues Pordeus, Benedito Barraviera, Rui Seabra Ferreira Junior, Vanessa Soares Lara, Karin Hermana Neppelenbroek, Heitor Marques Honório, Vinicius Carvalho Porto

**Affiliations:** 1 Department of Periodontics and Prosthodontics, Dental School of Bauru, FOB-USP—University of São Paulo, Al. Octávio Pinheiro Brisola, 9-75, Bauru 17012-901, Brazil; helenavenante@gmail.com (H.S.V.); anapaula.chappuis@gmail.com (A.P.C.-C.); rodrigomoreira@usp.br (R.M.B.d.C.); maripordeus@gmail.com (M.D.P.); vcporto@fob.usp.br (V.C.P.); 2 School of Dentistry, Universidad Espíritu Santo, Samborondon, Guayas 09-01-952, Ecuador; drmarcilo.oscar@gmail.com; 3 Department of Surgery, Stomatology, Pathology and Radiology, Dental School of Bauru, FOB-USP—University of São Paulo, Al. Octávio Pinheiro Brisola, 9-75, Bauru 17012-901, Brazil; rafaela@fob.usp.br (R.A.d.S.); vanessa@fob.usp.br (V.S.L.); 4 Department of Infectology, Dermatology, Imaging Diagnosis and Radiotherapy, Botucatu Medical School, FMB, São Paulo State University, UNESP, Av. Prof. Mário Rubens Guimarães Montenegro, Botucatu 18610-307, Brazil; bbviera@gmail.com (B.B.); rui.seabra@unesp.br (R.S.F.J.); 5 Center for the Studies of Venoms and Venomous Animals, CEVAP, São Paulo State University, UNESP, Av. Universitária, 3780, Fazenda Lageado, Botucatu 18610-307, Brazil; 6 Department de Pediatric Dentistry, Orthodontics and Public Health, Dental School of Bauru, FOB-USP—University of São Paulo, Al. Octávio Pinheiro Brisola, 9-75, Bauru 17012-901, Brazil; heitorhonorio@usp.br

**Keywords:** *Candida albicans*, denture stomatitis, complete denture, acrylic resin, CAD-CAM

## Abstract

The characteristics of the denture base surface, in combination with the oral environment, promote the colonization and development of *Candida albicans* biofilm, which is the main cause of denture stomatitis. This study evaluated the effectiveness of fibrin biopolymer with digluconate chlorhexidine or *Punica granatum* alcoholic extract to prevent *C. albicans* biofilm. Conventional heat polymerized and pre-polymerized poly(methyl methacrylate) (PMMA) circular specimens (10 × 2 mm) were fabricated (*n* = 504) and randomly divided into groups: no treatment (control—CT), fibrin biopolymer coating (FB), fibrin biopolymer with *P. granatum* (FBPg), or digluconate of chlorhexidine (FBCh) coating. The specimens were inoculated with *C. albicans* SC5314 (1 × 10^7^ cells/mL) and incubated for 24, 48, and 72 h. Crystal violet and colony-forming unit assays were used to quantify the total biofilm biomass and biofilm-living cells. A qualitative analysis was performed using confocal laser scanning microscopy. Data obtained are expressed as means and standard deviations and were statistically analyzed using a three-way analysis of variance (α = 0.05). The FBPg and FBCh groups inhibited the growth of *C. albicans* biofilm in both PMMA materials analyzed, with FBCh performing better in all periods evaluated (*p* < 0.0001). The colony forming unit (CFU) assay showed that the FB group favored the *C. albicans* biofilm growth at 24 h and 48 h (*p* < 0.0001), with no differences with CT group at 72 h (*p* = 0.790). All groups showed an enhancement in biofilm development up to 72 h (*p* < 0.0001), except the FBCh group (*p* = 0.100). No statistical differences were found between the PMMA base materials (*p* > 0.050), except in the FB group (*p* < 0.0001). Fibrin biopolymer, albeit a scaffold for the growth of *C. albicans,* when combined with chlorhexidine digluconate or *P. granatum*, demonstrated excellent performance as a drug delivery system, preventing and controlling the formation of denture biofilm.

## 1. Introduction

Denture stomatitis (DS) is a chronic inflammatory condition that affects 15% to 70% of denture wearers and is mainly related to inflammation caused by *Candida albicans* of the palatal mucosa supporting the denture [1,2,3]. *C. albicans* can colonize and develop a biofilm on the inner surface of the dentures, a process influenced by the hydrophobicity, surface free energy, and surface roughness of the poly(methyl methacrylate) (PMMA) resins [4,5].

The adhesion of *C. albicans* to the denture base is a critical factor in the development of DS [6,7]. This adhesion occurs through the colonization of microorganisms to the denture surface. These act as protective reservoirs that inhibit Candida from being removed by the self-cleaning effect of saliva, mechanical cleaning, or dislodgment forces [8]. This yeast has significant proliferation capacity, especially for immunocompromised patients [9].

Conventional heat-cured PMMA resin is the most widely used material for denture bases, and it has been proven that the processing method used increases the porosity of the acrylic surface [10]. In addition to conventional PMMA, newer processing systems for obtaining denture bases have been reported, such as computer-aided design and computer-aided manufacturing (CAD-CAM) PMMA-based polymers [11,12,13]. CAD-CAM-fabricated complete dentures have several promising advantages, including a decrease in porosity as the polymer base is formed from a pre-polymerized block of acrylic resin industrially polymerized under protocol conditions at high heat and pressure [13,14,15]. Moreover, denture bases fabricated using CAD-CAM release a small amount of monomer, which may affect microbial adhesion [16]. The internal surface of the denture bases in both PMMA processing methods are not polished, which may affect the roughness threshold, favoring *Candida* biofilm adhesion [17,18].

Besides reinforcing oral hygiene, DS treatment usually involves the administration of local and systemic antifungals [2,19,20]. However, these approaches may have certain setbacks such as local and systemic side effects, and antifungal resistance, which can lead to a disease recurrence [2,20]. Denture cleansers and oral mouth rinses are also used as antimicrobial agents; for the management and control of DS, however, their effectiveness depends directly on their continuous and proper use, following the manufacturer’s recommendations on the preparation and immersion period [21,22,23]. In addition, their use has been associated with increased surface roughness and the color changes of the base of the denture, which consequently favors the biofilm development [22,23]. Thus, it is necessary to develop new alternatives to promote anti-adherent and antimicrobial activity on PMMA substrates.

In this context, a growing interest in medicinal plants has been recently observed, and their therapeutic and preventive effects have been reported, including for DS [2,24,25]. The pomegranate (*Punica granatum*) is a fruit that is commonly consumed fresh or in beverages and has high antioxidant activity [25]. Furthermore, its alcoholic extract has already been proven to possess antifungal activity against *Candida* species [2,19,25,26,27]. Almeida et al. [2] incorporated in vitro anti-*C. albicans*, an alcoholic extract of *P. granatum,* into a denture adhesive. Furthermore, a study by Esawy et al. [28] showed that mouthwashes with fractions of *P. granatum* extract were effective as anti-calculus and anti-hemorrhagic agents.

In addition, coating materials have also been suggested as an alternative to promote changes in the topography and physicochemical characteristics of the PMMA, resulting in it being less prone to the adhesion and growth of *C. albicans*, such as synthetic tissue adhesives, specifically cyanoacrylates (CA) [1,4,29,30]. However, the high cost and controversial biocompatibility of CA continue to be issues [31].

More recently, a new heterologous fibrin sealant composed of fibrinogen-rich cryoprecipitate, extracted from *Bubalus bubalis* buffalo blood, and a thrombin-like enzyme, purified from the venom of *Crotalus durissus terrificus,* has been considered as a promising material in medicine and dentistry [32,33,34,35,36]. This biopolymer has biocompatibility (nontoxic), low-cost to produce in addition to hemostatic, sealant, adhesive, scaffold, and drug delivery properties [32,33,34,35,36,37,38,39]. In the presence of calcium, the thrombin-like enzyme acts on fibrinogen molecules, turning them into fibrin monomers, resulting in a stable clot [33,34,35]. In dentistry, Barbosa et al. [35,40] and Chiquito et al. [41] reported that using FB (fibrin biopolymer) to immobilize free gingival grafts is as effective as conventional sutures. In addition, it promoted advanced healing and reduced inflammatory cell density. However, its application as a coating material for complete dentures such as a drug delivery system has never been used.

According to the literature, the incorporation of antimicrobial agents into coating materials has demonstrated promising results, providing clinical advantages as a useful strategy to avoid microbial colonization [42,43,44]. Redding et al. [42] evaluated the effectiveness of incorporating chlorhexidine diacetate, amphotericin B, and nystatin into a thin-film polymer. Although all antifungals promoted satisfactory biofilm inhibition, the chlorhexidine diacetate group presented significantly better results. Therefore, considering the lack of studies and the necessity of new alternatives to treat and prevent DS, the aim of this study was to evaluate for the first time the effectiveness of a new heterologous fibrin sealant incorporated with digluconate chlorhexidine or *P. granatum* alcoholic extract against *C. albicans* biofilm using two different PMMA base materials. The null hypothesis was that the different coating conditions would not affect the formation of *C. albicans* biofilm.

## 2. Materials and Methods

### 2.1. Plant Material and Extract Preparation

*P. granatum* fruit were purchased at the “Boa Fruta” fruit and seedling distributor supermarket. The fruit was cultivated in Petrolina, Pernambuco, Brazil (9°4630′ S, 24°2130′ W). For this study, crude powder extract produced from the peel of the *P. granatum* fruit was used. The peels were dried in an air circulation oven at 45 °C (Drying Kiln with Renovation/Air Circulation Marconi N480 Novus; Marconi Ltda, Piracicaba, Brazil) and crushed in a knife mill (MA340—Macro Moinho de Willey Knives; Marconi Ltda, Piracicaba, Brazil) [2]. For the extraction of its compounds, percolation was performed at room temperature. This produced a hydroalcoholic extract [2]. The concentrated solution was subjected to a rotary evaporator (Bath Model Hei Vap Precision G3; Heidolph, Schwabach, Germany) under reduced pressure and at temperatures below 50 °C to remove the organic solvent. Finally, the crude extract was lyophilized in its powder form [25,45].

### 2.2. Specimen Preparation

A total of 504 circular specimens (10 mm diameter × 2 mm thick) using a pre-polymerized resin (Vipi block gum; Vipi, Pirassununga, Brazil) and heat polymerized acrylic resin (Vipi Cril Plus; Vipi, Pirassununga, Brazil) were prepared in accordance with the manufacturer’s instructions. To obtain a highly rough surface compatible with the growth of microorganisms (2–3 µm range), each specimen had one surface sanded using a polishing machine (PFL; Fortel, São Paulo, Brazil) with 120-grit sandpaper (Norton Abrasivos, Guarulhos, Brasil) for 15 s [30,46]. Surface roughness was measured using a roughness tester (Hommel Tester T 1000 basic; Hommelwerke GmbH, Vs-Schwenningen, Germany), and four readings were taken at different positions on the roughened surface of each specimen [47]. Subsequently, the specimens were immersed in 2 mL of distilled water at 37 °C for 48 h to allow for the release of residual monomer (ISO 10139-2, International Organization for Standardization, 1999) and were sterilized using ethylene oxide (Acecil Ltda, Campinas, Brazil) [47,48]. Finally, the specimens were randomly distributed into four groups: without coating or control group (CT), coated with FB only, coated with biopolymer fibrin incorporated with chlorhexidine (FBCh) or *P. granatum* (FBPg).

### 2.3. Surface Treatment of the Specimens

The fibrin biopolymer was discovered, developed and supplied by the Center for the Study of Venoms and Venomous Animals from São Paulo State University (CEVAP), Botucatu, São Paulo, Brazil. CEVAP maintains a serpentarium with authorization for the management of wild fauna (no. 3507.7263/2012-SP) and registration as a scientific breeder for research purposes at the Brazilian Institute of the Environment and Natural Resources (IBAMA) (protocol number 02001.005670/90–77). The extraction of the venom from *Crotalus durissus terrificus* snakes is carried out following the strictest standards of good laboratory practices and standard operating protocols to guarantee the quality and purity required in the production of biopharmaceuticals [49]. First, the venom is extracted and filtered, then the processing is carried out by evaluating its protein dosage. The venom undergoes a lyophilization process and then fractionated through high-performance liquid chromatography (HPLC), where the purity of the thrombin-like enzyme is evaluated by sequencing techniques and mass spectrometry [50,51]. Next, the fraction is stored at −20 °C. More information about the fibrin biopolymer is available on YouTube at https://youtu.be/y6ho6M0amA8 (accessed on 22 March 2021).

*Bubalus bubalis buffaloes* are used for the large-scale production of cryoprecipitate [37]. To guarantee that the cryoprecipitate is safe and free of any foreign or detrimental substance to the human organism, donor animals are selected and their health is evaluated. The animals are submitted to frequent sanitary management (vaccines and deworming, diagnostic serological tests, and tuberculinization tests, besides clinical examinations). CEVAP researchers extract the cryoprecipitate, which is then analyzed by two-dimensional electrophoresis, to isolate and identify proteins. The research bases for processing and traceability were reported by Pontes et al., 2017 [52] and Ferreira Jr. et al., 2019 [53]. The specifications of formulation are protected by patents no. BR 10 2014 011432 7 and BR 10 2014 011436-0 [54,55].

The concentrations of the tested antimicrobials that were incorporated into the fibrin biopolymer were established according to a pilot study and corresponded to 20 mg/mL of *P. granatum* and 4 mg/mL of chlorhexidine (data not shown). The biopolymer is available in three solutions: fraction 1 (0.400 mL)—*Crotalus durissus terrificus* thrombin-like enzyme, fraction 2 (1 mL)—buffalo cryoprecipitate solution, and diluent (0.600 mL)—containing calcium chloride. These compounds were kept frozen and thawed prior to use. The amounts used were 0.400 mL of fraction 1, 0.400 mL of diluent, and finally, 0.200 mL of fraction 2, which should be the last to be homogenized, since it is the responsible for polymerization, totaling 1 mL. Then, the powdered medicines were incorporated into the biopolymer until a homogeneous mixture was formed.

The experimental specimens were coated with 50 µL of each experimental product, which was equally distributed across the surface using a disposable brush tip (Disposable Brush Tips/60; 3M ESPE, Sumaré, Brazil). Afterward, the specimens were dried at room temperature for 40 min.

### 2.4. Yeast Strain, Growth Conditions and Biofilm Development

*C. albicans* (strain SC5413) frozen culture stocks (−80 °C) were incubated in tryptic soy broth (Accumidia manufactures Inc, Lansing, MI, USA) with 1% chloramphenicol (Quemicetina Succinato, Pfizer, Guarulhos, Brazil) at 30 °C for 24 h under aerobic conditions. Subsequently, the suspension was centrifuged at 5000 rpm for 10 min at 22 °C and the cells were harvested and washed with phosphate-buffered saline (PBS, pH 7.2) and standardized to 1 × 10^7^ cell/mL^−1^ in PBS using a hemocytometer [46,56].

To form the biofilm, all acrylic resin specimens were carefully washed with PBS and immersed in 1 mL of the previously standardized cell suspension and incubated for 90 min at 37 °C, at 75 rpm. Then, the specimens were washed in 1 mL of PBS to remove non-adherent organisms and immersed in 1 mL of Roswell Park Memorial Institute solution (RPMI-1640; Gibco, New York, NY, USA) for 24, 48, and 72 h at 37 °C (75 rpm). The medium was changed at 24-h intervals [2,46].

### 2.5. Viable Cell Count (Colony-Forming Units (CFU)/mL)

For this experiment, 108 specimens of each acrylic resin was fabricated. Each group included 3 specimens, and the experiment was repeated 3 times per period. After each evaluation period, the specimens were gently washed in 1 mL of PBS. The biofilm was removed from the specimen surface with the aid of a cell scraper (Costar^®^ 3010; Corning, New York, NY, USA) and stored in 1 mL of PBS [2,57]. These suspensions were serially diluted (10^−1^ to 10^−4^), and aliquots (50 µL) of each dilution were plated in triplicate on Sabouraud Dextrose agar (Accumedia Manufacturers, Lansing, MI, USA) and incubated for 48 h at 37 °C [58]. After this period, the colonies were counted and expressed as mean CFU/mL values.

### 2.6. Total Biomass of the C. albicans Biofilm

For this assay, 108 specimens of each acrylic resin were produced. Each group included 3 specimens, and the experiment was repeated 3 times per period. After the formation of the biofilm, the non-adherent cells were removed by washing the specimens in 1 mL of PBS in each well. Then, the biofilm formed on the rough surface was fixed with 1 mL of 99% methanol (Merck Millipore, Burlington, VT, USA) for 15 min, dried at room temperature, and immersed in 2 mL of 0.1% violet crystal (VC) solution (Sigma–Aldrich, St. Louis, MO, USA) for 20 min. The specimens were then washed with distilled water to remove the excess VC [59]. To dissolve the stain, the specimens were immersed in 2 mL of 95% ethanol (Synth, Diadema, Brazil) [60], and an aliquot of 100 µL was transferred to a 96 wells microtiter plate for spectrophotometer reading, programmed with a wavelength of 570 nm [46,61].

### 2.7. Confocal Laser Scanning Microscopy

After 24, 48, and 72 h of incubation, 36 specimens of each resin type were transferred to a sterile 24-well plate and carefully washed with PBS. In sequence, in the absence of light, the specimens were stained with LIVE/DEAD^®^ BaclightTM L7007 Kit (Molecular Probes; Sigma–Aldrich, St. Louis, MO, USA) at 1% for 20 min at 37 °C. The stained *C. albicans* biofilm remaining on the acrylic resin surface was qualitatively analyzed using confocal laser scanning microscopy (CLSM) (TCS-SPE; Leica Mycrosystems, Wetzlar, Germany) [1].

### 2.8. Statistical Analysis

Data obtained from the CFU and VC assays are presented as means and standard deviations and were statistically analyzed using a 3-way analysis of variance (α = 0.05).

## 3. Results

### 3.1. CFU Assay

Data from the CFU assay is shown in Table 1 and Table 2. The chlorhexidine group incorporated in the fibrin biopolymer (FBCh) exhibited high inhibitory values in all of the materials studied, showing statistically significant differences when compared with the CT, FB, and FBPg groups (*p* < 0.0001). In addition, it can be confirmed that this material has an inhibitory capacity of up to 72 h of exposure in microbial biofilm, with no significant differences between the periods evaluated (*p =* 0.100) in all of the materials studied (Table 1 and Table 2).

At 24 h, it was evident that the FBPg group was not statistically different (*p =* 0.060) than the control group in all of the materials studied. However, at 48 and 72 h, this group showed inhibitory capacity when compared to the control group (*p* < 0.0001) in all of the materials studied. FB favored *C. albicans* biofilm growth compared to the control group at 24 and 48 h in all of the materials evaluated (*p* < 0.0001). However, at 72 h, these differences were not significant (*p =* 0.790; Table 1 and Table 2).

Comparison among materials in the evaluation periods revealed significant differences between heat polymerized and CAD-CAM resins when FB coating was applied (*p =* 0.0100).

### 3.2. Metabolic Activity Test (VC)

Cellular accounting, expressed in absorbance values, is described in Table 3 and Table 4. Significant differences were observed for both the factors “groups” and “periods” (*p* < 0.0001). However, no statistical significance was found for the factor “material” (*p =* 0.830). Inhibitory effects were observed for the FBPg and FBCh groups when compared to the control group (*p* < 0.0001), with FBCh having the lowest values. However, there were no significant differences between the CT and FB groups (*p =* 0.590).

Regarding the factor “period”, a significant increase in the biofilm adhered to the surface was observed at 72 h in all the groups evaluated (*p* < 0.0001).

### 3.3. Qualitative Confocal Analysis

**Figure 1:**
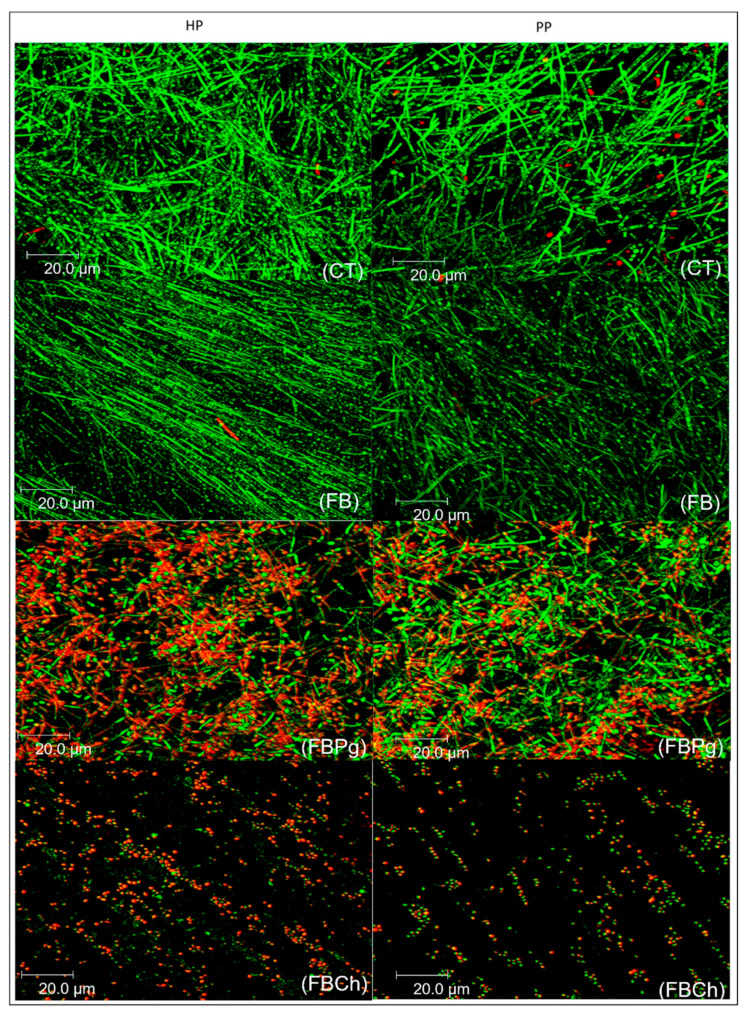
Confocal images of *C. albicans* biofilms developed on heat-polymerized (HP) and pre-pol ymerized (PP) surfaces after 24 h of incubation. CT: control group; FB: Fibrin biopolymer; FBPg: *P. granatum* incorporated in fibrin biopolymer; FBCh: Chlorhexidine incorporated in fibrin biopolymer.

After 24 h of incubation, the control counterpart showed a higher proportion of uniform and elongated *C. albicans* yeast arranged in clusters (Figure 1). Similarly, at the same time point, the FB group was densely populated with uniform yeast (Figure 1). In contrast, growth suppression of *C. albicans* by FBCh (Figure 1) and FBPg (Figure 1) was seen with LIVE/DEAD staining, confirming the CFU and VC assay data.

**Figure 2:**
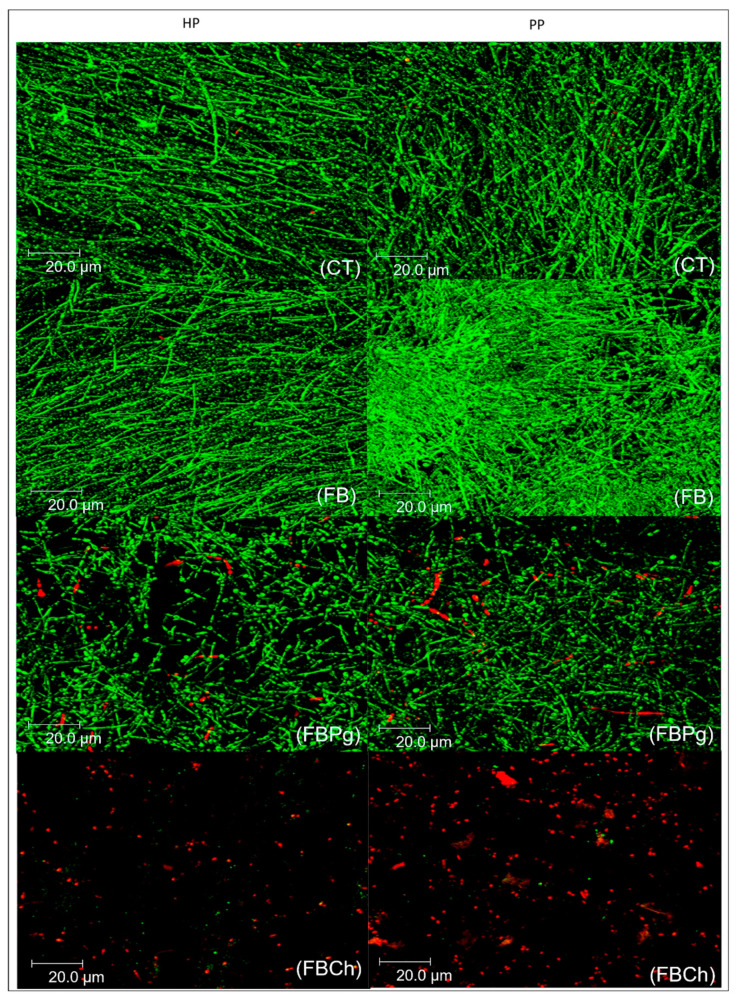
Confocal images of *C. albicans* biofilms developed on heat-polymerized (HP) and pre-polymerized (PP) surfaces after 48 h of incubation. CT: control; FB: Fibrin biopolymer; FBPg: *P. granatum* incorporated in fibrin biopolymer; FBCh: Chlorhexidine incorporated in fibrin biopolymer.

After 48 h of incubation, in the control (Figure 2) and FB groups (Figure 2), a large amount of yeast and hyphae were evident, ratifying the quantitative CFU and VC findings. Some *C. albicans* blastopores and yeasts were noted in biofilms treated with FBPg (Figure 2), while sparsely distributed *C. albicans* blastopores and dead yeast were detected in the FBCh group (Figure 2).

**Figure 3:**
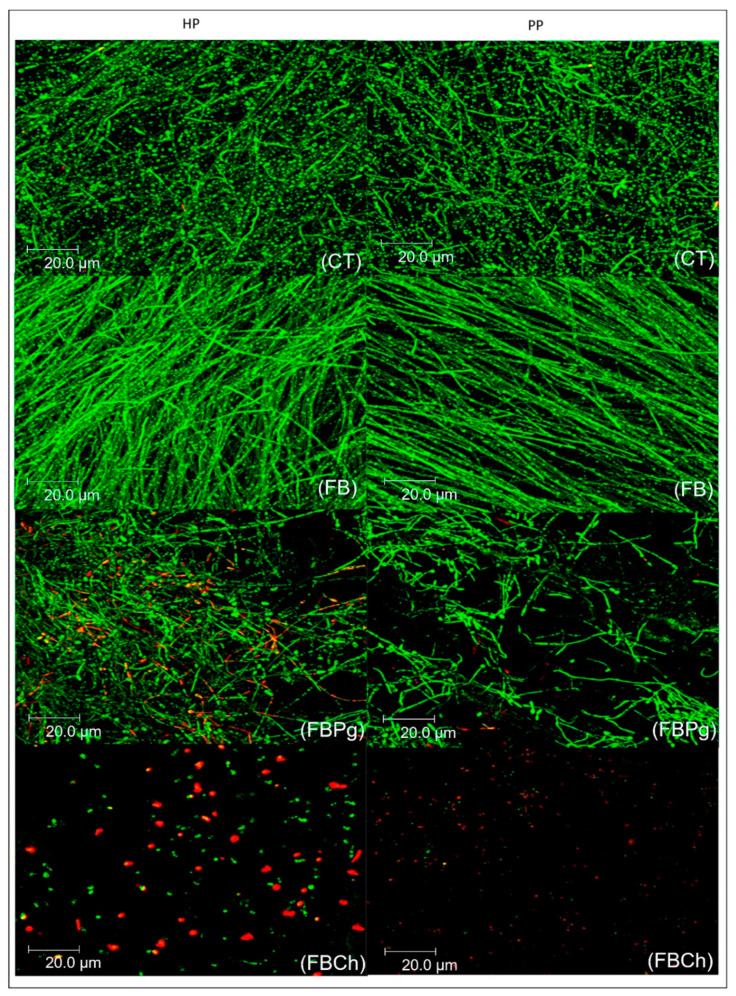
Confocal images of *C. albicans* biofilms developed on heat-polymerized (HP) and pre-polymerized (PP) surfaces after 72 h of incubation. CT: control group; FB: Fibrin biopolymer; FBPg: *P. granatum* incorporated in fibrin biopolymer; FBCh: Chlorhexidine incorporated in fibrin biopolymer.

After 72 h of incubation, the FB (Figure 3) and control groups (Figure 3) were highly densely populated with uniform yeast, pseudohyphae, and hyphae, similar to the results found in the CFU and VC assay. At the same time point, sparsely distributed *C. albicans* blastopores and a considerable amount of dead yeast were visible among scattered *C. albicans* in the FBCh group (Figure 3). Yeast and some hyphae of diffuse formats were evident in the group treated with FBPg (Figure 3).

## 4. Discussion

DS, the most common oral condition involving denture wearers, is mainly caused by the adherence of *C. albicans* to the porous and rough surfaces of denture base materials [7,8]. Coating the denture surface may alter these rough characteristics and prevent the colonization of this yeast. Strategies such as the incorporation of antimicrobials into the composition of coating materials can provide additional benefits in preventing the development of biofilms on the inner surface of removable dentures [42,43]. Thus, in the present study, we assessed whether the coating of fibrin biopolymer adhesive with *P. granatum* and digluconate chlorhexidine could affect the adhesion of *C. albicans* on the surface of two different PMMA resins.

To the best of our knowledge, no study has investigated fibrin biopolymer combined with antimicrobial agents nor its effectiveness against fungus as an alternative therapy for DS. The results showed that heat-treated and pre-polymerized resin specimens coated with fibrin biopolymer incorporated with chlorhexidine (FBCh) significantly reduced *C. albicans* biofilm formation and growth in all of the evaluated assays and periods. These findings are supported by Redding et al. [42] who evaluated chlorhexidine incorporated thin-film polymer on acrylic resin specimens after 24 h of incubation. In addition, Garaicoa et al. [44] showed the antifungal capacity of chlorhexidine incorporated in denture adhesives. Ellepola and Samaranayake [62] found that the chlorhexidine digluconate compound provoked rupture in the cell membrane yeast, even at low concentrations, and was a potent antifungal. Moreover, the antimicrobial efficacy of chlorhexidine is also associated with its substantivity, assuring its gradual release, and the promotion of its efficacy over a long period [63].

Although not as efficient as chlorhexidine, the incorporation of *P. granatum* in fibrin biopolymer (FBPg) also showed an inhibitory capacity on *C. albicans* biofilm at all periods and in both evaluated PMMA materials. Almeida et al. [2] also reported reduced biofilm development up to 12 h after biofilm induction in specimens treated with prosthetic adhesive with *P. granatum* incorporation. The antifungal effect of crude hydroalcoholic extract from *P. granatum* peel has been attributed to several structural compounds of the peel, specifically the punicalagin and ellagitannin derivatives [25,26,64]. These components are found in abundance in the crude hydroalcoholic extract of *P. granatum* peels, causing serious damage to the cellular structure of *C. albicans* yeast [26,27], possibly related to their molecular structure and toxicity, and astringent properties of tannins [45].

Surprisingly, and observed for the first time, FB favored biofilm development for at least 72 h in both quantitative tests. As far as we know, none of the studies before found these *C. albicans*-scaffold properties. Therefore, only indirect comparisons can be worthy of the previous discussion. Biofilm overgrowth could be related to the presence of abundant fibrin net—a special biological material—acting as a nutrient reservoir for *C. albicans* development on the resin surface [65]. However, it is noteworthy that this study proved the wet tolerance of FB and its efficiency as a drug delivery system for antimicrobials or antifungals. In this way, FBs incorporated with antimicrobials could be a sustainable alternative for the local prevention and management of DS. Moreover, previous studies have demonstrated the biocompatibility or absence of cytotoxicity of FB in human and animal cells [32,33,34]. It should be noted that for the first time, a biodegradable biological material (fibrin biopolymer) was innovatively applied together with antimicrobial-yeast candidate agents to prevent DS.

Among the two different PMMA materials, the CAD-CAM pre-polymerized resin presented the best inhibitory effect to the development of *C. albicans* biofilm. Recent studies have demonstrated that the surface of CAD-CAM specimens exhibit significantly less adherence to *C. albicans* than heat-cured specimens [11,14]. These results could be attributed to the surface roughness of the materials once the pre-polymerized PMMA presents a smoother surface compared to heat-cured resin [14,15].

Considering the time exposure evaluated in this investigation, all groups presented a significant overgrowth of *C. albicans* biofilm after 72 h, as observed in other *Candida* species-related studies [44]. The development of *C. albicans* biofilm proceeds in three developmental phases: early (0–11 h), intermediate (12–30 h), and maturation (38–72 h). In the present study, the overgrowth of *C. albicans* biofilm was detected in the mature phase of biofilm development, which is probably associated with its highly heterogeneous architecture and extracellular material [49], besides greater drug tolerance since mature biofilm starts to express resistance genes [3,59]. In addition, “persister” necrotic fungal cells in mature biofilm subjected to antifungal agents, which are subpopulations of cells highly tolerant of stress conditions, protected by the cell-matrix can repopulate the biofilm and interact and co-aggregate with other microorganisms present in the oral environment. This represents an important factor in its virulence [66,67].

In summary, the results of the present study suggested that fibrin biopolymer, a natural biological material, facilitates the growth of *C. albicans*, possibly due to the robust fibrin network. On the other hand, when this biological medicine was applied as a drug delivery system, associated with molecules that are candidates for antifungal agents, such as chlorhexidine or *P. granatum* used in this investigation, there was an important inhibition in dentures biofilm production. These outcomes suggest that the incorporation of *P. granatum* or digluconate chlorhexidine in a vehicle tolerant to a wet environment has the potential to prevent and treat DS by acting on the main etiological factor, *C. albicans.* However, among the limitations of this study, it comprised in vitro rather than in vivo tests, thus, these findings must be carefully applied to clinical conditions, since the adhesive will be subjected to routine hygiene and disinfection protocols performed by denture users, in addition to thermal and pH variations that may affect the effect and durability of this experimental product. Thus, further in vivo investigations on the anti-adherent potential against biofilm-associated with the denture base are needed to prove its effectiveness against DS, determine its longevity, and whether it causes any damage to the structure of dentures.

## 5. Conclusions

Within the limitations of this in vitro study and according to the data obtained, it was possible to conclude that, although fibrin biopolymer facilitated *C. albicans* growth, it proved to be effective when used as a drug delivery-system in combination with digluconate chlorhexidine or *P. granatum,* resulting in a reduction of fungal biofilm growth on the surface of two different PMMA base materials up to 72 h.

## Figures and Tables

**Table 1:** Mean values of colony forming unit (CFU)/mL (10^3^) ± standard deviations (SD) in heat polymerized resin.

Groups	Experimental Periods			Mean ± SD/Group
	24 h	48 h	72 h	
CT	1304.44 ± 500.07 Aa	1726.44 ± 386.45 Aa	3144.44 ± 474.11 Ba	2058.44 ± 453.55
FB	2297.78 ± 613.55 Ab	2557.78 ± 758.19 Ab	3220 ± 424.15 Ba	2691.85 ± 598.63
FBPg	891.11 ± 178.36 Aa	1277.78 ± 196.58 Ac	2186.67 ± 220.45 Bb	1451.85 ± 198.46
FBCh	0.0 ± 0.0 c	0.07 ± 0.2 d	0.02 ± 0.07 c	0.03 ± 0.09
Mean ± SD/Period	1123.33 ± 322.99 (10^3^)	1390.52 ± 335.35 (10^3^)	2137.78 ± 279.69 (10^3^)	

CT: Control group; FB: Fibrin biopolymer; FBPg: *P. granatum* incorporated in fibrin biopolymer; FBCh: chlorhexidine incorporated in fibrin biopolymer. Horizontally, different capital letters indicate a statistical difference between the experimental periods for the same group (*p* < 0.05). Vertically, different lowercase letters indicate a statistical difference between the groups for the same period (*p* < 0.05).

**Table 2:** Mean values of CFU/mL (10^3^) ± standard deviations (SD) in computer-aided design and computer-aided manufacturing (CAD-CAM) resin.

Groups	Experimental Periods			Mean ± SD/Group
	24 h	48 h	72 h	
CT	1271.11 ± 315.73 Aa	1504.44 ± 458.89 Aa	2664.44 ± 402.71 Ba	1813.33 ± 392.44
FB	1842.22 ± 410.18 Ab	1753.33 ± 597.99 Ab	3044,44 ± 434.14 Ba	2213.33 ± 480.77
FBPg	888.89 ± 251.62 Aa	1053.33 ± 190.79 Ac	1753.33 ± 410.97 Bb	1231.85 ± 284.46
FBCh	0.02 ± 0.07 c	0.02 ± 0.07 d	0.09 ± 0.2 c	0.04 ± 0.11
Mean ± SD/Period	1000.56 ± 244.39 (10^3^)	1077.78 ± 311.94 (10^3^)	1865.58 ± 312.01 (10^3^)	

CT: Control group; FB: Fibrin biopolymer; FBPg: *P. granatum* incorporated in fibrin biopolymer; FBCh: chlorhexidine incorporated in fibrin biopolymer. Horizontally, different capital letters indicate a statistical difference between the experimental periods for the same group (*p* < 0.05). Vertically, different lowercase letters indicate a statistical difference between the groups for the same period (*p* < 0.05).

**Table 3:** Mean absorbance values (nm) ± standard deviations (SD) of the metabolic activity of the *C. albicans* biofilm for the adhesives tested on specimens made with conventional heat polymerized resin.

Groups	Experimental Periods			Mean ± SD/Group
	24 h	48 h	72 h	
CT	1576.56 ± 850. 56	1897.67 ± 702.62	2763.444 ± 387.19	2079.23 ± 646.79 a
FB	1879.78 ± 702.88	2003.44 ± 776	2102.333 ± 1179.4	1995.18 ± 886.09 a
FBPg	1153.44 ± 647.59	1470.56 ± 591.57	1598.333 ± 672.35	1407.44 ± 637.17 b
FBCh	110 ± 2.74	120.11 ± 4.76	127.889 ± 5.71	119.33 ± 4.4 c
Mean ± SD/Period	1179.95 ± 550.94 A	1372.95 ± 518.74 A	1647.99 ± 561.16 B	

CT: Control group; FB: Fibrin biopolymer; FBPg: *P. granatum* incorporated in fibrin biopolymer; FBCh: chlorhexidine incorporated in fibrin biopolymer. Horizontally, different capital letters indicate a statistical difference between the experimental periods for the same group (*p* < 0.05). Vertically, different lowercase letters indicate a statistical difference between the groups for the same period (*p* < 0.05).

**Table 4:** Average absorbance values (nm) ± standard deviations (SD) of the metabolic activity of the *C. albicans* biofilm for the adhesives tested on specimens made with CAD-CAM resin.

Groups	Experimental Periods			Mean ± SD/Group
	24 h	48 h	72 h	
CT	1591.778 ± 583.75	1475.667 ± 612.7	2126.556 ± 1204.09	1731.33 ± 800.18 a
FB	1925.889 ± 438.85	1777.667 ± 685.53	2643.333 ± 340	2115.63 ± 488.13 a
FBPg	1500.778 ± 177.55	1330.222 ± 520.12	1807.111 ± 976.89	1546.04 ± 558.19 b
FBCh	136.222 ± 2.28	126 ± 14.16	141.333 ± 12.37	134.52 ± 9.6 c
Mean ± SD/Period	1288.67 ± 300.61 A	1177.39 ± 458.13 A	1679.58 ± 633.34 B	

CT: Control group; FB: Fibrin biopolymer; FBPg: *P. granatum* incorporated in fibrin biopolymer; FBCh: chlorhexidine incorporated in fibrin biopolymer. Horizontally, different capital letters indicate a statistical difference between the experimental periods for the same group (*p* < 0.05). Vertically, different lowercase letters indicate a statistical difference between the groups for the same period (*p* < 0.05).

## Data Availability

The data presented in this study are available on request from the corresponding author.
